# Phylogenetic placement of *Leprariacryptovouauxii* sp. nov. (Lecanorales, Lecanoromycetes, Ascomycota) with notes on other *Lepraria* species from South America

**DOI:** 10.3897/mycokeys.53.33508

**Published:** 2019-05-20

**Authors:** Beata Guzow-Krzemińska, Agnieszka Jabłońska, Adam Flakus, Pamela Rodriguez-Flakus, Magdalena Kosecka, Martin Kukwa

**Affiliations:** 1 Department of Plant Taxonomy and Nature Conservation, Faculty of Biology, University of Gdańsk, Wita Stwosza 59, PL-80-308 Gdańsk, Poland University of Gdańsk Gdańsk Poland; 2 Department of Lichenology, W. Szafer Institute of Botany, Polish Academy of Sciences, Lubicz 46, PL-31-512 Kraków, Poland W. Szafer Institute of Botany, Polish Academy of Sciences Kraków Poland; 3 Laboratory of Molecular Analyses, W. Szafer Institute of Botany, Polish Academy of Sciences, Lubicz 46, PL-31-512 Kraków, Poland Institute of Botany, Polish Academy of Sciences Kraków Poland

**Keywords:** lichenized fungi, morphology, Neotropics, nucITS rDNA, secondary metabolites, taxonomy

## Abstract

*Leprariacryptovouauxii* is described as a new semicryptic species similar to *L.vouauxii*, from which it differs geographically (South America) and phylogenetically; both species differ in nucleotide position characters in nucITS barcoding marker. *Leprariaharrisiana* is reported as new to South America and *L.nothofagi* as new to Antarctica, Bolivia, and Peru. *Leprariaincana* (South American records are referred to L.aff.hodkinsoniana) and *L.vouauxii* (most South American records are referred to *L.cryptovouauxii*) should be excluded at least temporarily from the lichen list of South America. All records previously referred to as *L.alpina* from Bolivia and Peru belong to *L.nothofagi*. Most of Bolivian records of *L.pallida* belong to *L.harrisiana*. *Leprariaborealis* and *L.caesioalba* should be included in *L.neglecta. Leprariaachariana*, *L.impossibilis*, and *L.sipmaniana* are sequenced for the first time.

## Introduction

Traditionally phenotypic characters have been used to separate lichen species; however, in numerous cases DNA based phylogenetic approaches suggested relationships that differ from traditional systematics. In some groups of lichens the absence of easily recognizable morphological or chemical characters in some lineages supported by phylogenetic signal lead to description of cryptic species (for discussion see Crespo and Perez-Ortega 2009; Crespo and Lumbsch 2010). Furthermore, Vondrák et al. (2009) introduced the semicryptic species concept for taxa that cannot be diagnosed based on their morphology but are determined based on their ecology and distribution. Moreover, for taxa with limited number of phenotypical characters due to the lack of sexual structures, the morphological species concept is especially challenging in discrimination of species (e.g., Lendemer 2011a, 2013a; Lendemer and Hodkinson 2013).

*Lepraria* Ach. (Lecanorales, Lecanoromycetes, Ascomycota) is a genus of crustose to fruticose lichen species, which during evolution apparently totally lost the ability of sexual reproduction and are always sterile (e.g., Ekman and Tønsberg 2002; Fehrer et al. 2008; Lendemer 2011a, 2013a; Lendemer and Hodkinson 2013). However, despite of that, they continued to speciate and 74 species are known worldwide (Ekman and Tønsberg 2002; Fehrer et al. 2008; Wijayawardene et al. 2017).

Thalli of *Lepraria* consist of soredia-like granules laying directly on a substrate or on a layer of hypothalline hyphae in case of crustose species or, in species with fruticose thalli, granules are produced also on short pseudopodetia (Lendemer 2011a; Lendemer and Hodkinson 2013). The edge of crustose thalli may be diffuse or obscurely to markedly lobate (Sipman 2004; Lendemer 2011a). Secondary chemistry is one of the most important characters in the determination and taxonomy of *Lepraria*, as morphological characters are scarce (e.g., Tønsberg 1992; Kukwa and Flakus 2009; Saag et al. 2009; Lendemer 2011a, 2013a). However, morphology and lichen substances must be taken under consideration together when identifying lichens of this genus as some species share the same (or very similar) morphologies or secondary chemistry (Tønsberg 1992; Leuckert et al. 1995; Elix and Tønsberg 2004; Sipman 2004; Flakus and Kukwa 2007, 2011; Fehrer et al. 2008; Kukwa and Flakus 2009; Saag et al. 2009; Lendemer 2013a, b; Lendemer and Hodkinson 2013).

Until recently the taxonomy of the genus has been based almost solely on morphological features and the content of secondary metabolites, and it included only those species having crustose thalli with elobate margins and lacking dibenzofurans (Laundon 1989, 1992; Tønsberg 1992). Subsequent studies demonstrated that *Leproloma* Nyl. ex Cromb. should also be included in *Lepraria* (Ekman and Tønsberg 2002; Kukwa 2002). On the other hand, *Leprarialesdainii* (Hue) R.C.Harris was transferred to the newly established genus *Botryolepraria* Canals et al. (Canals et al. 1997), which appeared to be more closely related to Verrucariaceae in Eurotiomycetes (Kukwa and Pérez-Ortega 2010) and *Leprariamoroziana* Lendemer and *L.obtusatica* Tønsberg to *Andreiomyces* Hodkinson & Lendemer (Hodkinson and Lendemer 2013) within Arthoniomycetes. Also, few fruticose species previously belonging to *Leprocaulon* Nyl. have been transferred to *Lepraria* as well, but few *Lepraria* species to *Leprocaulon* and other genera (Grube et al. 2004; Bungartz et al. 2013; Lendemer and Hodkinson 2013). The status of several other species remains unsettled, especially of those containing usnic acid, as no molecular data are available for some taxa and their phylogenetic position has not yet been determined (Kukwa 2006a; Osyczka et al. 2010; Fryday and Øvstedal 2012; Orange et al. 2017).

In this paper we present new molecular data on *Lepraria* based on specimens collected in South America. Three species have been sequenced for the first time and sequences of other species made possible to clarify status of some taxa in Bolivia and other South American countries. *Leprariacryptovouauxii* is described as new to science.

## Material and methods

### Taxon sampling

The studied specimens from South America and Antarctica are deposited in the following herbaria: C, KRAM, LPB, S, and UGDA. The measurements of thallus structures of the new species were taken in water, often with addition of ethanol. This procedure, used by Olszewska et al. (2014), reduced the hydrophobic properties of lichen substances present in the thallus and made all structures easier to observe. Ethanol was selected as it did not affect the size of the granules, which was empirically tested. The secondary chemistry of all samples was studied by thin layer chromatography (TLC) following methods by Orange et al. (2001). Confirmation of identified substances was achieved in some cases by running the extracts adjacent to an extract containing known substances.

**Figure 1.**
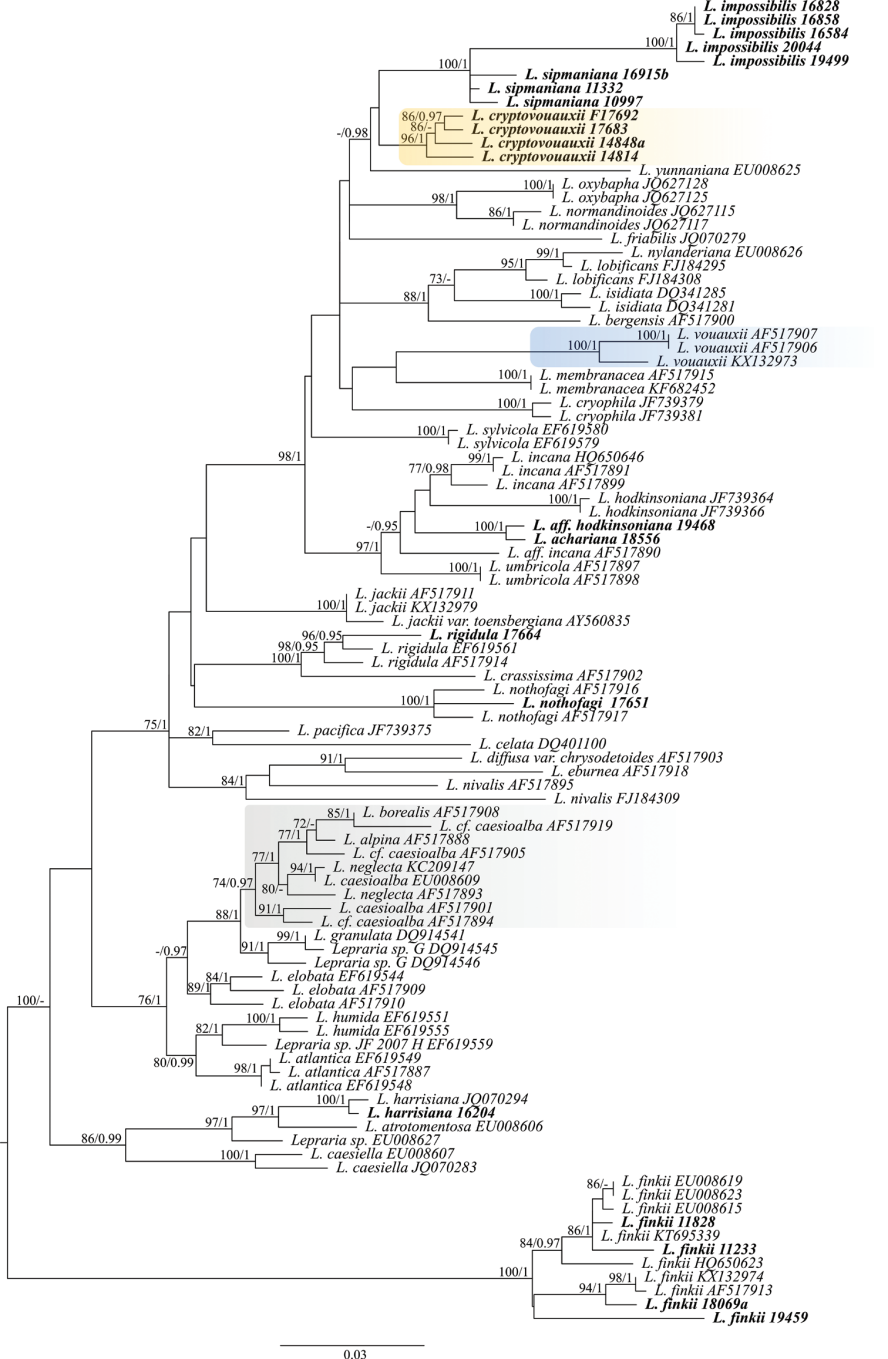
ML tree based on nucITS rDNA dataset for *Lepraria* spp. with midpoint rooting. Newly sequenced specimens of *Lepraria* are in bold and their names are followed with collection number of specimens. In case of the sequences obtained from GenBank the taxa names are followed with accession numbers. Bootstrap supports from ML analysis ≥ 70 (first value) and posterior probabilities from BA ≥ 0.95 (second value) are indicated near the branches. The newly described *L.cryptovouauxii* is highlighted in orange, *L.vouauxii* is highlighted in blue, and *L.neglecta* is highlighted in grey.

In addition, specimens of *Leprarianothofagi* Elix & Kukwa reported as *Lepraria* sp. 1 and sequenced by Ekman and Tønsberg (2002) were reinvestigated and their sequences, along with other sequences of *Lepraria* spp., were downloaded from GenBank. Their accession numbers are given in Figure 1. In the preliminary analysis we used all available sequences of *Lepraria* spp. for the alignment which was further reduced to representatives of each species. We excluded from the dataset very short sequences, and numerous identical or very similar sequences. Finally, each species or clade is represented by at least one or two representatives, except *L.neglecta* (Nyl.) Erichsen and *L.finkii* (de Lesd.) R.C.Harris that were better sampled due to their high variation in nucITS rDNA marker. Sequences of *L.lobificans* Nyl. non auct. were originally named as *L.santosii* Argüello & A.Crespo, but the latter name was synonymized with *L.lobificans* by Lendemer (2013a), and therefore the last name was used in Figure 1.

### *DNA extraction, PCR amplification, and DNA sequencing*

Specimens were selected after detailed analyses of morphology and chemistry and only uncontaminated samples were used for molecular studies. Samples of all *Lepraria* species reported from South America by Flakus and Kukwa (2007), Flakus et al. (2011a), and Kukwa and Flakus (2009) were subjected to DNA extraction and sequencing, but nucITS rDNA sequences were obtained for only nine species. We assumed that DNA degraded in samples collected more than three years prior to DNA extraction procedure.

DNA was extracted using a modified CTAB method (Guzow-Krzemińska and Węgrzyn 2000) or the E.Z.N.A.SP Fungal DNA Kit (Omega Bio-tek, Inc.) following manufacturer’s protocol.

Fungal nucITS rDNA was amplified using the following primers ITS1F (Gardes and Bruns 1993) and ITS4A (Kroken and Taylor 2001) or ITS5 and ITS4 (White et al. 1990). The same primers were used for sequencing.

PCR was carried out in a volume of 25 µl using Color Perpetual *Taq* DNA Polymerase (Eurx) or StartWarm HS-PCR Mix (A&A Biotechnology) following the manufacturer’s protocols. In each case 2 or 3 µl of genomic DNA was used for amplification. The following PCR cycling parameters were applied to amplify nuclear ITS region: an initial denaturation at 94 °C for 3 min, followed by 35 cycles at 94 °C for 30 s, 54 °C for 30 s (for ITS1F and ITS4 primers) or 52 °C for 30 s (for ITS5 and ITS4 primers), and 72 °C for 1 min, with a final extension at 72 °C for 10 min. In case of ITS1F and ITS4A primers the following parameters were used: an initial denaturation at 94 °C for 2 min, followed by 35 cycles at 94 °C for 30 s, 60 °C for 1 min, and 72 °C for 1 min, with a final extension at 72 °C for 7 min.

PCR products were visualized on 1% agarose gels stained with ethidium bromide or SimplySafe (Eurx) dyes in order to determine DNA fragment lengths. Subsequently, PCR products were purified using High Pure PCR Product Purification Kit (Roche Diagnostic GmbH) or Wizard SV Gel and PCR Clean-Up System (Promega).

The purified PCR products were sequenced using Big Dye Terminator v. 3.1 Cycle Sequencing Kit (Applied Biosystems) and primers as listed above: 1 µl of Big Dye Terminator, 2 µl of sequencing buffer, 0.5 µl DMSO with 1.5 µl (1 M) of primer, 1–4 µl of amplified products and dd H_2_O in a total reaction volume of 10 µl. Cycle sequencing profile in Eppendorf thermal cycler consisted of: 20 s of initial denaturation, followed by 25 cycles, each with denaturation at 94 °C for 15 s, annealing at 52 °C for 20 s and elongation at 60 °C for 4 min. The sequences were read in Genomed (Warsaw). Alternatively, the sequencing was performed in Macrogen (the Netherlands/South Korea). The DNA sequences were assembled and manually adjusted in Auto Assembler v. 1.4.0 (Parker 1997) and SeaView v. 4.1 (Galtier et al. 1996; Gouy et al. 2010). The newly obtained sequences were deposited in GenBank database and their accession numbers are listed in Table 1.

**Table 1.** List of *Lepraria* specimens newly sequenced for this study with their nucITS rDNA GenBank Accession numbers. All samples were collected in Bolivia.

Species	Voucher	GenBank Accession No.
* L. achariana *	Kukwa 18556 (UGDA)	MK629283
* L. cryptovouauxii *	Flakus 17683, Rodriguez (KRAM)	MK629272
	Flakus 17692, Rodriguez (KRAM)	MK629270
	Kukwa 14848a, holotype (UGDA)	MK629273
	Flakus 14814, Rodriguez (KRAM)	MK629271
* L. finkii *	Kukwa 11233 (UGDA)	MK629288
	Flakus 11828, Kukwa (KRAM)	MK629285
	Kukwa 18069a (UGDA)	MK629287
	Kukwa 19459 (UGDA)	MK629286
* L. harrisiana *	Kukwa 16204 (UGDA)	MK629284
L. aff. hodkinsoniana	Kukwa 19468 (UGDA)	MK629282
* L. impossibilis *	Kukwa 16584 (UGDA)	MK629279
	Kukwa 16828 (UGDA)	MK629281
	Kukwa 16858 (UGDA)	MK629280
	Kukwa 19499 (UGDA)	MK629278
	Flakus 20044, Quisbert (KRAM)	MK629277
* L. nothofagi *	Flakus 17651, Rodriguez (KRAM)	MK629268
* L. rigidula *	Flakus 17664, Rodriguez (KRAM)	MK629269
* L. sipmaniana *	Kukwa 10997 (UGDA)	MK629274
	Kukwa 11332 (UGDA)	MK629275
	Kukwa 16915b (UGDA)	MK629276

### Sequence alignment and phylogenetic analysis

The newly generated nucITS rDNA were compared to the sequences available in the GenBank database (http://www.ncbi.nlm.nih.gov/BLAST/) using BLASTn search (Altschul et al. 1990). The alignment was generated using Seaview software (Galtier et al. 1996; Gouy et al. 2010) employing muscle option followed with Gblocks selection of poorly aligned sites using less stringent parameters (Castresana 2000). In the final dataset, we analyzed 97 sequences of different *Lepraria* spp. The final alignment consisted of 546 unambiguous sites of which 282 were constant.

Moreover, all available sequences of *Leprariacryptovouauxii* and *L.vouauxii* were aligned using Seaview software (Galtier et al. 1996; Gouy et al. 2010) employing muscle option and followed with trimming of terminal ends. The final alignment consisted of seven sequences and 515 sites. Then variable sites were selected and presented in Table 2. Finally, we chosen those nucleotide position characters from alignment that support the distinction of *L.cryptovouauxii* (Table 2).

**Table 2.** Variable positions in the alignment of *Leprariacryptovouauxii* (marked in bold) and *L.vouauxii.* First sequence, i.e. KX132973 is treated as a reference sequence and dots represent nucleotides identical to reference sequence. Diagnostic nucleotide position characters in the fungal barcoding marker, nucITS, to distinguish *L.cryptovouauxii* from *L.vouauxii* are highlighted in yellow.

Position in the alignment/ Species															1	1	1	1	1	1	1	1	1	1	1
	1	1	2	2	3	3	4	6	6	8	8	8	9	9	0	0	1	1	2	2	3	4	5	5	6
	0	4	7	8	8	9	0	3	4	1	7	8	2	4	0	6	8	9	3	4	3	9	0	7	5
* L. vouauxii * KX132973	C	G	C	T	C	C	T	T	G	G	C	C	T	C	C	C	C	T	C	C	G	A	C	G	G
* L. vouauxii * AF517906	.	.	.	C	T	T	.	.	.	.	.	T	C	.	.	.	.	C	.	T	.	.	.	.	.
* L. vouauxii * AF517907	.	.	.	C	T	T	.	.	.	.	.	T	C	.	.	.	.	C	.	T	.	.	.	.	.
***L.cryptovouauxii* 17692**	**A**	.	**T**	**A**	**T**	.	.	**G**	**A**	**A**	**T**	**T**	.	**T**	**T**	**A**	.	**A**	**A**	.	.	.	**A**	**T**	**A**
***L.cryptovouauxii* 14814**	**A**	.	**T**	**C**	**T**	.	.	**G**	**A**	**A**	**T**	**T**	.	**T**	**T**	**A**	**A**	**A**	**A**	.	**A**	.	**A**	**T**	**A**
***L.cryptovouauxii* 17683**	**A**	**T**	**T**	**A**	**T**	.	.	**G**	**A**	**A**	**T**	**T**	.	**T**	**T**	**A**	.	**A**	**A**	.	.	.	**A**	**T**	**A**
***L.cryptovouauxii* 14848a**	**A**	.	**T**	**A**	**T**	.	**C**	**G**	**A**	**A**	**T**	**T**	.	**T**	**T**	**A**	.	~**A**	**A**	.	.	**C**	**A**	**T**	**A**
**Position in the alignment/ Species**	**1**	**1**	**1**	**1**	**1**	**1**	**1**	**3**	**3**	**3**	**4**	**4**	**4**	**4**	**4**	**4**	**4**	**4**	**4**	**4**	**4**	**4**	**4**	**4**	**5**
	**7**	**7**	**8**	**8**	**8**	**8**	**9**	**3**	**3**	**3**	**1**	**3**	**4**	**6**	**6**	**7**	**7**	**7**	**7**	**7**	**8**	**8**	**9**	**9**	**1**
	**0**	**1**	**5**	**6**	**7**	**8**	**1**	**2**	**4**	**8**	**1**	**0**	**5**	**0**	**3**	**1**	**2**	**3**	**4**	**9**	**5**	**7**	**3**	**5**	**4**
* L. vouauxii * KX132973	G	T	G	T	C	A	G	C	T	A	G	G	G	A	G	A	C	A	C	C	T	G	C	T	C
* L. vouauxii * AF517906	.	.	.	.	.	.	.	.	C	.	A	.	.	.	C	.	.	.	.	.	.	.	T	.	.
* L. vouauxii * AF517907	.	.	.	.	.	.	.	.	C	.	A	.	.	.	C	.	.	.	.	.	.	.	T	.	.
***L.cryptovouauxii* 17692**	**A**	**G**	.	.	.	.	**A**	**A**	.	**G**	.	**T**	**T**	**T**	**T**	.	**A**	**C**	**A**	**G**	**A**	.	**T**	**A**	**A**
***L.cryptovouauxii* 14814**	**A**	**G**	**T**	.	**A**	**C**	**A**	**A**	.	**G**	.	**T**	**T**	**T**	**T**	**G**	**A**	.	**A**	**G**	**A**	.	**T**	**A**	**A**
***L.cryptovouauxii* 17683**	**A**	**G**	.	.	**A**	.	**A**	**A**	.	**G**	.	**T**	**T**	**T**	**T**	.	**A**	**C**	**A**	**G**	**A**	.	**T**	**A**	.
***L.cryptovouauxii* 14848a**	**A**	**G**	.	**G**	**A**	.	**A**	**A**	.	**G**	.	**T**	**T**	**T**	**T**	.	**A**	.	**A**	**G**	**A**	**A**	**T**	**A**	**A**

We used Partition Finder 2 (Lanfear et al. 2016) implemented at CIPRES Science Gateway (Miller et al. 2010) to determine the best substitution model for each partition under Akaike Information Criterion (AIC) and greedy search algorithm (Lanfear et al. 2012). Two different models were found for two partitions, i.e. K80+I for 5.8S and GTR+G+X for ITS1 and ITS2 regions. The phylogenetic analyses were performed using Markov Chain Monte Carlo (MCMC) as implemented in MrBayes v. 3.2.2 (Huelsenbeck and Ronquist 2001; Ronquist and Huelsenbeck 2003) at CIPRES Science Gateway (Miller et al. 2010). The dataset was analysed employing K80+I and GTR+G+X models for 5.8S and ITS partitions with 10 M generations, 2 independent runs, each with four chains. The output of MrBayes was analyzed with the program Tracer v. 1.5 (Rambaut and Drummond 2007) and the initial 25% of trees were discarded as burn-in and the majority-rule consensus tree was calculated to obtain posterior probabilities (PP).

Maximum likelihood (ML) analyses were performed using RaxML HPC v. 8 on XSEDE (Stamatakis 2014) under the GTRGAMMAI model at CIPRES Science Gateway (Miller et al. 2010). Rapid bootstrap analyses were performed with 1000 bootstrap replicates.

The phylogenetic trees were drawn using FigTree v. 1.4.2 (Rambaut 2009). RaxML bootstrap support (BS values ≥ 70) and PP values (values ≥ 0.95) are given near the branches on phylogenetic tree.

The alignments and trees are deposited at TreeBASE database under accession 24193.

### Haplotype networks

Sequences of nucITS rDNA marker from specimens of the newly described *Leprariacryptovouauxii* as well as *L.impossibilis* Sipman and *L.sipmaniana* (Kümmerl. & Leuckert) Kukwa were aligned using Seaview software (Galtier et al. 1996; Gouy et al. 2010) and the terminal ends were trimmed. The alignment consisted of 12 sequences and 530 sites.

The nucITS rDNA sequences of *Leprariafinkii* downloaded from GenBank were aligned together with newly generated sequences of this species using Seaview software (Galtier et al. 1996; Gouy et al. 2010) and terminal ends were deleted. The final alignment consisted of 21 sequences and 464 sites.

For both datasets TCS networks (Clement et al. 2002) were created with 95% connection limit and gaps treated as missing as implemented in PopART software (http://popart.otago.ac.nz).

## Results and discussion

Twenty-one new nucITS rDNA sequences were generated from nine *Lepraria* species for this study (Table 1). Among them *Leprariaachariana* Flakus & Kukwa, *L.impossibilis*, and *L.sipmaniana* as well as the newly described *L.cryptovouauxii* (see taxonomic part) were sequenced for the first time.

Based on nucITS rDNA dataset, topologically congruent trees were generated using maximum likelihood method (ML; best tree likelihood LnL = −5906.670489) and Bayesian approach (BA; harmonic mean was −5936.28). In Bayesian analysis, the average standard deviation of split frequencies was 0.002901 and the average PSRF for parameter values was 1.000. The ML tree was presented in Figure 1 with added bootstrap supports (BS) from ML analysis and posteriori probabilities (PP) from BA.

The newly sequenced specimens collected in Bolivia were resolved in different clades within the phylogenetic tree of *Lepraria* (Fig. 1). Five sequences of *L.impossibilis* form a highly supported clade (100 in ML and 1 in BA), which is closely related to *L.sipmaniana* represented by three newly sequenced specimens (which however do not form a well-supported group), *L.cryptovouauxii* represented by four sequenced specimens forming a well-supported clade (96 in ML and 1 in BA) and one sequence of *L.yunnaniana* (Hue) Zahlbr. All those species, except *L.yunnaniana* which contains divaricatic acid, produce pannaric acid 6-methylester.

**Figure 2.**
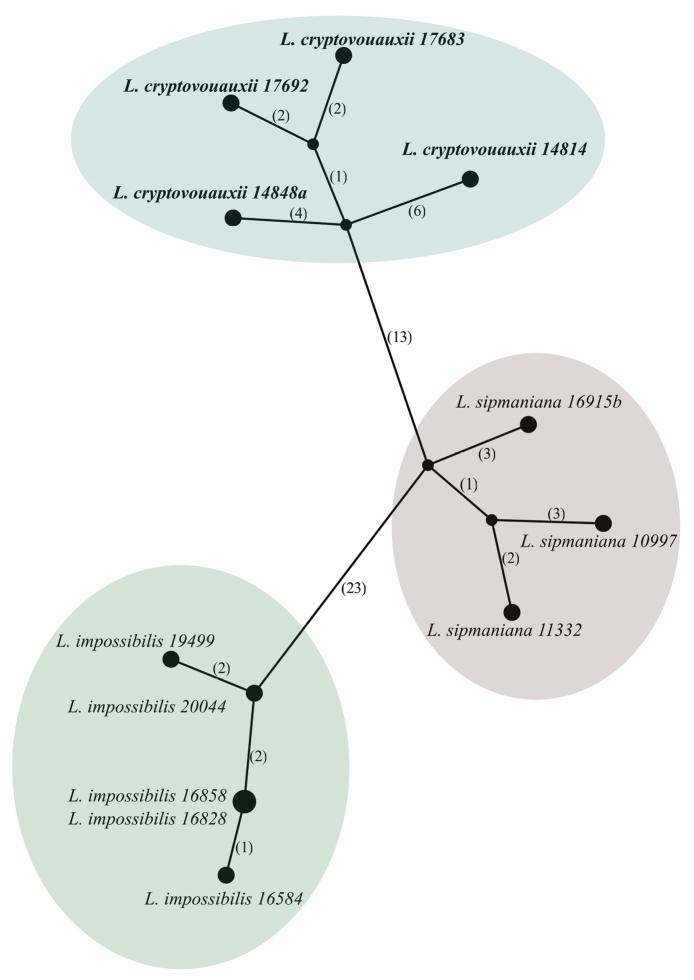
Haplotype network showing relationships between nucITS rDNA sequences from selected *Lepraria* spp. Newly generated nucITS rDNA sequences from *L.cryptovouauxii*, *L.impossibilis* and *L.sipmaniana* were analyzed. The names of species are followed with herbarium numbers of specimens. Mutational changes are presented as numbers in brackets near lines between haplotypes. Haplotypes corresponding to each of species are highlighted with separate elipses. The newly described *L.cryptovouauxii* is given in bold.

To better understand phylogenetic position and genetic variation of nucITS rDNA marker within group of taxa containing pannaric acid 6-methylester, we generated haplotype network for specimens of all three species (Fig. 2). *Leprariacryptovouauxii* differs in at least 19 positions from *L.sipmaniana* and 39 positions from *L.impossibilis*. This analysis showed that each of the species is well separated from others; however, we observed some infraspecific variation. In our dataset the haplotypes of newly described *L.cryptovouauxii* differ in at least seven to 10 mutational steps from each other and in case of *L.sipmaniana* in five to seven steps. The lowest variation was found in *L.impossibilis* for which two specimens share the same nucITS rDNA haplotype while other haplotypes differ in one to five positions from each other. Our study showed that nucITS rDNA marker is variable in this group of species at the infra and interspecific levels.

*Leprariacryptovouauxii* was previously assigned to *L.vouauxii* (Hue) R.C.Harris as it shares secondary chemistry and very similar morphology with the latter (Flakus and Kukwa 2007; Flakus et al. 2011a). Molecular data, however, have shown that four South American specimens with obscurely lobate thalli containing pannaric acid 6-methylester and thus assignable to *L.vouauxii* represent a very different taxon forming a separate clade unrelated to the three sequences of *L.vouauxii* obtained from European specimens (Ekman and Tønsberg 2002; Mark et al. 2016). *Leprariavouauxii* is resolved in a highly supported clade (100 in ML and 1 in BA (Fig. 1). Moreover, their nucITS rDNA sequences differ in numerous positions of which some may be used as diagnostic characters to distinguish those taxa (Table 2). *Leprariacryptovouauxii* and *L.vouauxii* can be treated as semicryptic species (Vondrák et al. 2009; Lendemer 2011a) as they differ in the distribution ranges (see the taxonomic part). Based on the new results, we assume that *L.vouauxii* should be at least temporarily excluded from the South American list of lichens (Table 3), but its occurrence there is not improbable (see the taxonomic part).

**Table 3.** Records of *Lepraria* from South America revised in this paper. Some samples of taxa marked with asterisk still need to be revised to clarify their identity (for more data see under each species).

Previously	In this paper
L.alpina*	* L. nothofagi *
* L. borealis *	* L. neglecta *
* L. caesioalba *	* L. neglecta *
* L. incana *	L. aff. hodkinsoniana
L.pallida*	*L.harrisiana* and *L.pallida*
L.vouauxii*	* L. cryptovouauxii *

*Leprariaimpossibilis* was described by Sipman (2004) as having lobate thallus, lobes with raised marginal rim, and producing pannaric acid 6-methylester and lecanoric acid. Flakus and Kukwa (2007) and Kukwa and Flakus (2009) assigned to this species also samples with thalli having diffuse margins. For our molecular analyses we used specimens with diffuse and lobate thalli and they all clustered together in a highly supported clade (100 in ML and 1 in BA) confirming that the morphology of *L.impossibilis* may vary and that the unique secondary chemistry is a diagnostic character.

*Leprariahodkinsoniana* Lendemer was described to accommodate the material containing divaricatic acid and zeorin, which was previously referred to as *L.incana* (L.) Ach. in North America. Due to that, the latter species was excluded from the list of North American lichens (Lendemer 2011a). *Leprariaincana* was also reported from South America (Flakus and Kukwa 2007; Flakus et al. 2011a, b, 2015); however, after the description of *L.hodkinsoniana*, we doubted it can represent the former species. Therefore, we sequenced one specimen morphologically and chemically consistent with the description of this species, but the new sequence appeared to be more closely related to the sequences of *L.hodkinsoniana* than to those of *L.incana* obtained from European specimens (Ekman and Tønsberg 2002; Schmull et al. 2011). However, the inclusion of the sequence of *L.achariana* (this species contains lecanoric acid as the main secondary metabolite) to the data set revealed that the latter forms a highly supported clade (100 in ML and 1 in BA) with Bolivian specimen similar to *L.hodkinsoniana* (Fig. 1). Due to that, we decided to name the Bolivian material with divaricatic acid and zeorin as L.aff.hodkinsoniana and additionally we propose to exclude *L.incana* from the list of South American lichens (Table 3). Whether the specimen of L.aff.hodkinsoniana is only a chemotype of *L.achariana* or represents another semicryptic species with chemistry similar to *L.incana*, cannot be solved now and more specimens of both, *L.achariana* and L.aff.hodkinsoniana, need to be sequenced. Sequence named as L.aff.incana (GenBank Acc. no. AF517890; Fig. 1) was originally assigned to *L.incana* (Ekman and Tønsberg 2002), but after the inclusion of *L.achariana* and *L.hodkinsoniana* to the data set, it is clear that this specimen may represent yet undescribed taxon and the whole group requires further studies.

Sample of *L.rigidula* (B. de Lesd.) Tønsberg collected in Bolivia clustered together with other samples of this species from Norway and Ukraine obtained from GenBank (Ekman and Tønsberg 2002; Fehrer et al. 2008) and was found to be closely related to *L.crassissima* (Hue) Lettau with high support (100 in ML and 1 in BA) (Fig. 1). This finding confirms the occurrence of *L.rigidula* in South America (Flakus and Kukwa 2007; Flakus et al. 2015).

*Leprarianothofagi* has been described from *Nothofagus* bark in Argentina (Flakus et al. 2011a). Here it is reported as new to Antarctica, Bolivia, and Peru, and for the first time, it is reported from rocks and terricolous bryophytes. The samples from Antarctica were previously included in the phylogeny of *Lepraria* by Ekman and Tønsberg (2002) as *Lepraria* sp. 1. The re-examination of those specimens revealed the chemistry characteristic for *L.nothofagi* (atranorin, strepsilin, and porphyrilic acid). The two sequences from those specimens form a highly supported clade (100 in ML and 1 in BA) with newly obtained sequence of *L.nothofagi* from Bolivia. That latter sample (Fig. 4D) was initially determined as *L.alpina* (B. de Lesd.) Tretiach & Baruffo, but the re-examination of the chemistry and morphology of this specimen as well as all other samples reported from South America by Flakus and Kukwa (2007) and Flakus et al. (2011a) revealed that they represent *L.nothofagi* (see the taxonomic part). Flakus and Kukwa (2007) have already pointed that the South American specimens of *L.alpina* studied by them had more powdery appearance than those examined from Europe. Moreover, according to Lendemer (2013b), sequences of samples with aggregate thalli containing porphyrillic acid, and thus referable to *L.alpina*, are nested within the *L.neglecta* group (Fig. 1), and the name is treated as a synonym of *L.neglecta* (Lendemer 2013a, b). Sequences representing members of the *L.neglecta* group are named in their original version in Figure 1; however, all those names are synonymous with *L.neglecta* (Lendemer 2013a, b). Because of that, *L.borealis* Lohtander & Tønsberg reported from Chile by Flakus et al. (2011a) and *L.caesioalba* (B. de Lesd.) J.R.Laundon reported from South America by Flakus and Kukwa (2007) and Flakus et al. (2011a) should be excluded from the lists of lichens occurring in South America and placed as synonyms of *L.neglecta* (Table 3).

*Leprariaharrisiana* Lendemer is reported in this paper as new to South America. The specimen of *L.harrisiana* used in phylogenetic analyses was at first assigned to *L.pallida* Sipman to which it is chemically similar in producing atranorin, zeorin, and fatty acids (Sipman 2004; Lendemer 2012). However, molecular data placed this specimen in the same highly supported clade (100 in ML and 1 in BA) with *L.harrisiana* from North America. Revision of Bolivian material previously assigned to *L.pallida* by Flakus and Kukwa (2007) and Flakus et al. (2011a, 2015) revealed that most Bolivian specimens of this species belong to *L.harrisiana* (Table 3), but one sample represents *L.pallida* s. str. (see taxonomic part).

**Figure 3.**
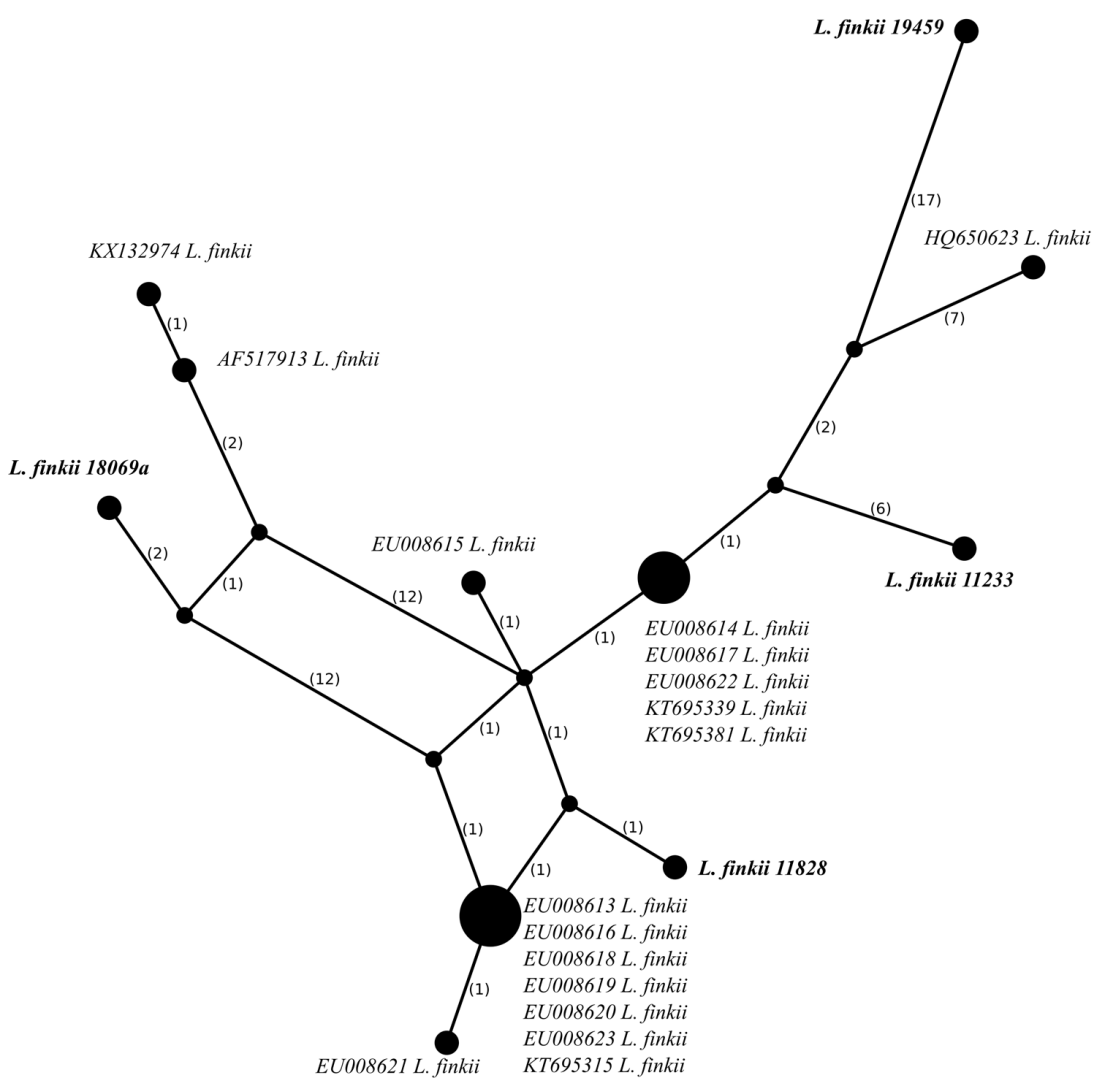
Haplotype network showing relationships between nucITS rDNA sequences from *Leprariafinkii*. Newly generated nucITS rDNA sequences are given in bold. The names of species are followed with herbarium numbers of specimens or accession numbers precede species names in case of sequences obtained from Genbank. Mutational changes are presented as numbers in brackets near lines between haplotypes.

Additionally, four specimens of *L.finkii* from Bolivia were sequenced and were found to belong to a highly supported clade (100 in ML and 1 in BA) together with *L.finkii* specimens from GenBank (Fig. 1). The specimens resolved in this clade were collected in different geographical areas, i.e. South America (Bolivia), North America (Canada and USA), and Europe (Norway and Switzerland). This clade clusters together genetically highly variable specimens. Haplotypes of *L.finkii* from Bolivia are unique and significantly differ from each other and other haplotypes (Fig. 3). The haplotype identified in specimen Kukwa 19459 differs from other known haplotypes in at least 24 mutational steps. The haplotype identified in specimen Kukwa 18069a is most similar to European records (Norway and Switzerland), from which it differs in five or six sites, respectively. Sequences from specimens Kukwa 11233 and Flakus 11828 are most similar to North American haplotypes (Canada and USA), from which they differ in at least seven or two positions, respectively. Those Bolivian specimens may represent cryptic taxa; however, this requires further study, which is beyond the scope of this paper.

**Figure 4.**
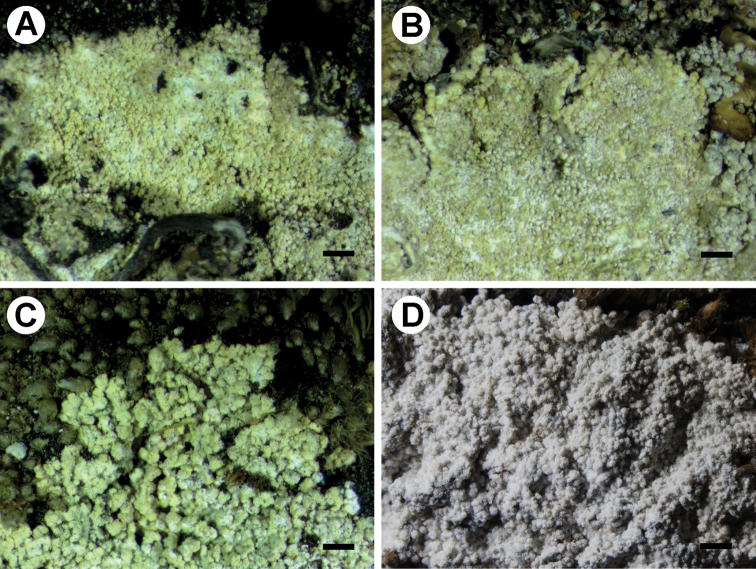
Morphology of *Leprariacryptovouauxii* (**A−C**) and *L.nothofagi* (**D**). **A** Holotype (M. Kukwa 14848a) **B** Thallus with obscurely lobate margins (Flakus 14814) **C** Thallus with large and compacted aggregations of granules (Flakus 17682) **D** Details of thallus (Flakus 17651 & Rodriguez). Scale bars: 500 µm (**A−C**), 300 µm (**D**).

### Taxonomy

#### Lepraria cryptovouauxii

Taxon classificationFungiLecanoralesStereocaulaceae

Kukwa, Flakus & Guzow-Krzemińska sp. nov.

830289

Fig. 4A–C

##### Diagnosis.

Species very similar to *Leprariavouauxii*, but differing in the distinct phylogenetic position within the genus (Fig. 1), in substitution of several nucleotide positions in nucITS (Table 2) and the occurrence in high altitudes of the Andes in South America.

##### Type.

Bolivia. Dept. La Paz; Prov. Franz Tamayo, Área Natural de Manejo Integrado Nacional APOLOBAMBA, road Pelechuco-Keara, 14°41'23"S, 69°08'02"W, elev. 4370 m, open high Andean vegetation, terricolous, 17 Nov. 2014, M. Kukwa 14848a (holotype UGDA, isotype LPB).

##### Description.

Thallus crustose, continuous, leprose, placodioid, up to 0.4 mm thick, distinctly grey-yellow, orange-yellow to brownish orange in colour; crisped margins absent, but some parts obscurely lobate; prothallus disappearing with age; hypothallus as layer of densely intertwined hyphae, hyphae hyaline, c. 3 μm wide; rhizohyphae present, brown pigmented, 3–3.5 μm wide; granules globose or subglobose, 20–70 μm in diameter, discrete, ecorticate, with outer part consisting of incomplete layer of hyphae (c. 3 μm wide) and incrusted with irregular groups of crystals insoluble in K, granules often forming compound units up to 100 μm in diameter (in one sample, Flakus 17682, up to c. 300 μm, Fig. 4C).

Photobiont green, coccoid, cells globose to subglobose, 5–11 μm.

##### Chemistry.

Pannaric acid-6-methylester (+, major), 4-oxypannaric acid-6-methyl ester (+, minor), vouauxii unknown 1 sensu Tønsberg (1992) (±, trace) and rarely traces of anthraquinones and atranorin (only in Flakus 8673).

##### Habitat and distribution.

*Leprariacryptovouauxii* grows on soil, rocks, or terricolous and saxicolous bryophytes in open and dry to moderately humid habitats at elevations between c. 3350 and 4790 m a.s.l.

Molecular data are available only for four Bolivian samples, but, judging on the basis of the ecological characteristic of other specimens and the altitudes they were collected in South America, we assume that *L.cryptovouauxii* occurs also in Chile, Ecuador, and Peru; the previous terricolous, muscicolous, or saxicolous records from the high Andes in South America belong here (Laundon 1989; Leuckert and Kümmerling 1991; Flakus and Kukwa 2007; Flakus et al. 2011a, 2015).

Few specimens with the same chemistry and similar morphology were collected on wood and tree bark (Flakus 7872, 8381; see Flakus and Kukwa 2007, Flakus 18440 and Kukwa 16829 collected in Dept. Tarija) are excluded from specimen list of *L.cryptovouauxii* due to the different habitat (cloud forests) and lower altitudes (up to c. 2300 m a.s.l.) on which they were collected. They may represent *L.vouauxii* or another undescribed taxon, but their nucITS sequences have not been obtained yet.

##### Etymology.

The name refers to the similarity in morphology and secondary chemistry to *Leprariavouauxii*.

##### Additional specimens examined.

BOLIVIA. Dept. La Paz: Prov. Bautista Saavedra, Área Natural de Manejo Integrado Nacional Apolobamba, between la Curva and Charazani, 15°08'09"S, 69°02'03"W, 3780 m alt., open area with shrubs, terricolous, 15 Nov. 2014, M. Kukwa 14675 (LPB, UGDA); Prov. Franz Tamayo, Área Natural de Manejo Integrado Nacional Apolobamba, near Puyo Puyo village, 14°56'55"S, 69°07'58"W, 4795 m alt., high Andean open vegetation, terricolous, 5 July 2010, A. Flakus 17683, 17692, P. Rodriguez (KRAM, LPB); Prov. Manco Kapac, Horca del Inca Mt. near Copacabana village, 16°10'15"S, 69°05'05"W, 3974 m alt., 18 June 2006, A. Flakus 8671.1, 8673 (KRAM, LPB); Prov. Murillo, near Cumbre pass, Puna, 16°19'18"S, 68°04'42"W, 4450 m alt., 17 June 2006, A. Flakus 8593.1 (KRAM, LPB, UGDA); Prov. Murillo, near Cumbre pass, Puna, 4672 m alt., 16°20'14"S, 68°02'20"W, 13 May 2006, A. Flakus 5729, 5730, 5731, 5733, 5738, 5740 (KRAM, LPB); ibidem, alt. 4604 m, 16°21'59"S, 68°02'37"W, 13 May 2006, A. Flakus 5791, 5798 (KRAM, LPB); near Cumbre pass, 4550 m alt., 16°19'18"S, 68°04'42"W, high Andean Puna vegetation, on mosses, June 2006, A. Flakus 8584.1, 8586, 8597.1 8600, 8603, 8605, 8606 (KRAM, LPB, UGDA); Prov. Omasuyos, El Dragon hill near Chahualla, 15°51'17"S, 69°00'40"W, 3850 m alt., Puna Húmeda vegetation, saxicolous, 6 July 2010, A. Flakus 17812, P. Rodriguez (KRAM, LPB); Dept. Potosí: Prov. Nor Lípez, Pinturas Rupestres near Villamar Mallcu village, 21°46'20"S, 67°29'05"W, 4038 m alt., open semi-desert high Andean area, terricolous, 6 Dec 2009, A. Flakus 14814, P. Rodriguez (KRAM, LPB). Chile. Terr. Magallanes, Lago del Toro (L. Maravilla), Estancia Río Payne, above the river, on soil, 15 March 1941, R. Santesson 6594 (S). Ecuador. Prov. León: Railway station Cotopaxi, alt. 3550 m, on bare soil in Páramo, 26 Apr 1939, E. Asplund L 63 (S). PERU. Dept. Ancash: Prov. Huaraz, Huaraz, 3500 m alt., on soil, 22 Nov 1972, C. de Graaf (UGDA); Dept. Arequipa: Prov. Caylloma, near Cabanaconde village, semi-desert open mountain area, 3462 m alt., 15°37'56"S, 71°57'49"W, terricolous, 2006, A. Flakus 9531, 9532, 9533, 9644 (KRAM); Valle del Colca, above Tapay village, open mountain area, alt. 3705 m, 15°33'56"S, 71°55'32"W, terricolous, 2006, A. Flakus 9692, 9693, 9766 (KRAM); near Socorro village, 3349 m alt., 15°38'32"S, 71°43'22"W, terricolous, 2006, A. Flakus 9416, 9419 (KRAM); between Soro and Llahuar villages, 15°34'41"S, 72°01'01"W, 2100 m alt., open semi-desert montane area, on soil and bryophytes over rocks, 6 July 2008, A. Flakus 10135, 10139, M. Kukwa 6107, 6108 (KRAM, UGDA); Dept. Cuzco: prov. Urubamba, valley of Rio Piri, NW of Ollantaytambo, 13°06'S, 72°22'W, 3400 m alt., on soil, 23 March 1981 R. Santesson P86: 17 (S); Dept. Lima: Prov. Huarochiri, valley of Rio Santa Eulalia, NE of Carampoma, 11°38'S, 76°27'W. c. 3700 m alt., on bryophytes, 15 Feb 1981, R. Santesson P24: 5, R. Moberg (S); Dept. Junin: Prov. Tarma, c. 10 km (road distance) NNE of Palca, 11°18'S, 75°32'W, c. 2600 m alt., on soil, 7 Feb 1981, R. Santesson P12: 60, R. Moberg (S – specimen of *Leprariadiffusa*).

##### Selected specimens of *Leprariavouauxii* examined for comparison.

Canada. Canadian Arctic Archipelago: Ellesemere I., Eureka, East Wind Lake, 80°05'N, 85°37'W, on terricolous mosses, 31 July 1999, F. Daniels s.n. (UGDA L-15825). Italy. Umbria: Monte Corona, vicinity of Eremo dell’Assunta Incoronata, 700 m alt., on rock, Jan 2001, A. Zwolicki s.n. (UGDA L-10052); ibidem, on *Quercus* sp., Jan 2001, A. Zwolicki s.n. (UGDA L-10148). Poland. Pojezierze Iławskie: Szymbark, Teutonic castle, 53°38'38"N, 19°28'57"E, on brick, 4 July 2003, J. Boczkaj, M. Kukwa s.n. (UGDA L-10020); Bory Dolnośląskie: Przewóz, on brick, 14 Sept 2000, Š. Bayerová et al. (UGDA L-10720). Ukraine. Opilya: Ivano-Frankivsk region, Halych district, Kosova Hora near Burshtyn, 49°13'25,7"N, 24°42'07,6"E, 300 m alt., steppe vegetation, on gypsum, 27 June 2003, L. Śliwa 1991 (UGDA L-11320).

##### Notes.

*Leprariacryptovouauxii* and *L.vouauxii* are practically indistinguishable in morphology and secondary chemistry. The only difference we could observe is the colour of thallus, which is more intensively orange-yellow in *L.cryptovouauxii*, while *L.vouauxii* tends to be more greyish green. However, Lendemer (2013a) mentioned similar more distinctly coloured specimens for *L.vouauxii* in material from North America. Whether those samples represent another yet undescribed species has not been resolved. The brighter colour observed in *L.cryptovouauxii* can be caused by a higher concentration of dibenzofurans which may act as a sunscreen in the very sunny habitats of the Andes. Similar tendency was observed also for *L.diffusa* (J.R.Laundon) Kukwa, in which thalli were more intensively coloured in sunny places in comparison to samples from shaded situations (Kukwa 2006b). Dibenzofurans, when in high concentration, can have a colour visible on TLC plates (before spraying with sulphuric acid), which suggest that they may play a sunscreen role as other pigmented substances and determine the colour of thallus (Kukwa 2006b).

According to Lendemer (2013a), *L.vouauxii* lacks brown rhizohyphae, which are present in the new species. To confirm this character as a possible discriminating feature, several specimens of *L.vouauxii* were studied to check the colour of rhizohyphae. We found that, similar to *L.cryptovouauxii*, rhizohyphae can be brown, but in some specimens they were very sparse, in some formed well-visible layer between the thallus and substrate, but some thalli lacked those hyphae. Tønsberg (1992) also mentioned the presence of brown rhizohyphae (as hypothallus). Apparently, *L.vouauxii* shows variation in the development and colour of this structure.

Despite the lack of morphological and chemical differences, *L.cryptovouauxii* can be distinguished on the basis of its distribution as it occurs in the high Andes in South America, whereas *L.vouauxii* remains unconfirmed from South America and genetically known only from Europe (Fig. 1; Ekman and Tønsberg 2002; Mark et al. 2016). The habitat preferences also differ to some extent as *L.cryptovouauxii* grows only on soil, rocks, or saxicolous and terricolous bryophytes, whereas *L.vouauxii* occurs on various substrates, including tree bark, rocks, and soil (Tønsberg 1992; Kukwa 2006b; Flakus and Kukwa 2007; Lendemer 2013a).

*Leprariadiffusa* is morphologically somewhat similar and also produces dibenzofurans; however, it has aggregate thallus (sensu Lendemer 2011b), and it produces oxypannaric acid-2-methylester (Laundon 1989; Leuckert and Kümmerling 1991; Leuckert et al. 1995; Elix and Tønsberg 2004; Lendemer 2013a).

*Leprariaxerophila* Tønsberg is the species which also contains pannaric acid-6-methylester as the major secondary metabolite, but it differs in placodioid thalli with crisped margins (Tønsberg 2004; Lendemer 2011b, 2013a). This metabolite is present also in some chemotypes of *L.tenella* (Tuck.) Lendemer & B.P. Hodk. (syn. *Leprocaulontenellum* (Tuck.) Nyl.), but this species differs in the almost constant production of lecanoric acid (at least always present together with pannaric acid-6-methylester) and atranorin and the development of pseudopodetia (Lamb and Ward 1974; Bungartz et al. 2013; Lendemer and Hodkinson 2013).

#### Lepraria harrisiana

Taxon classificationFungiLecanoralesStereocaulaceae

Lendemer

##### Remarks.

Most Bolivian records (except one cited below) of *L.pallida* presented in Flakus and Kukwa (2007) and Flakus et al. (2011a, 2015) were revised and belong to *L.harrisiana*. Here only the new record is presented.

##### Specimens of examined.

Bolivia. Dept. Chuquisaca: Prov. Zudañez, Área Natural de Manejo Integrado El Palmar, La Cascada bajo de El Palmar, 18°41'23"S, 64°54'26"W, 2740 m atl., Boliviano-Tucumano forest with *Podocarpus*, Lauraceae and palms, corticolous, 15 July 2015, M. Kukwa 16204 (LPB, UGDA).

#### Lepraria aff. hodkinsoniana

Taxon classificationFungiLecanoralesStereocaulaceae

Lendemer

##### Remarks.

All known Bolivian records of *L.incana* presented in Flakus and Kukwa (2007), and Flakus et al. (2011a, b, 2015) should be assigned to L.aff.hodkinsoniana. Here two new records are presented.

##### Specimens of examined.

Bolivia. Dept. Cochabamba: Prov. Carrasco, Parque Nacional Carrasco, Meruvia, 17°34'59"S, 65°15'06"W, 3215 m alt., upper montane Yungas forest, corticolous, 4 Nov. 2016, M. Kukwa 18041 (LPB, UGDA); Dept. Santa Cruz: Prov. Comarapa, Parque Nacional y Área Natural de Manejo Integrado Amboró, Remate, 17°51'39"S, 64°21'15"W, 2270 m alt., natural Yungas forest, on dead tree fern, 15 May 2017, M. Kukwa 19468 (LPB, UGDA).

#### Lepraria nothofagi

Taxon classificationFungiLecanoralesStereocaulaceae

Elix & Kukwa

##### Remarks.

Records of *L.alpina* from Bolivia and Peru (Flakus and Kukwa 2007; Flakus et al. 2011a) and *Lepraria* sp. 1 from Antarctica (Ekman and Tønsberg 2002) belong to *L.nothofagi*. Here only new record is presented.

Some other records of *L.alpina* (Flakus et al. 2011a) should be revised to assess if they represent *L.neglecta* or *L.nothofagi*.

##### Specimens examined.

Bolivia. Dept. La Paz; Prov. Franz Tamayo, Área Natural de Manejo Integrado Nacional APOLOBAMBA, near Puyo Puyo village, 14°56'55"S, 69°07'58"W, 4795 m alt., high Andean open vegetation, on bryophytes, 5 July 2010, A. Flakus 17651 & P. Rodriguez (KRAM, LPB).

#### Lepraria pallida

Taxon classificationFungiLecanoralesStereocaulaceae

Sipman

##### Remarks.

This is the only so far known Bolivian record of this species.

Some other records from Brazil and Peru (Flakus and Kukwa 2007; Flakus et al. 2011a) still need to be revised.

##### Specimens of examined.

Bolivia. Dept. La Paz; Prov. Nor Yungas, near Pacallo village, 16°12'10"S, 67°50'39"W, 1360 m alt., Yungas montane forest, on rocks and saxicolous bryophytes, 3 Aug. 2008, M. Kukwa 7172 (LPB, UGDA).

## Supplementary Material

XML Treatment for Lepraria cryptovouauxii

XML Treatment for Lepraria harrisiana

XML Treatment for Lepraria aff. hodkinsoniana

XML Treatment for Lepraria nothofagi

XML Treatment for Lepraria pallida
